# Impact of Nebulized BromAc^®^ on Mucus Plug Clearance in a Mechanically Ventilated Ex Vivo Ovine Lung Model of Obstructive Respiratory Conditions

**DOI:** 10.3390/life14091111

**Published:** 2024-09-03

**Authors:** Nicole Valle, Mathew Suji Eapen, Krishna Pillai, Richard Morris, Javed Akhter, Ahmed H. Mekkawy, David L. Morris, Sarah J. Valle

**Affiliations:** 1 Mucpharm Pty Ltd., Sydney, NSW 2217, Australia; nicole@mucpharm.com (N.V.); krishna@mucpharm.com (K.P.); javed@mucpharm.com (J.A.); ahmed@mucpharm.com (A.H.M.); david@mucpharm.com (D.L.M.); 2 Intensive Care Unit, Shoalhaven District Memorial Hospital, Nowra, NSW 2541, Australia; richard.morris@heath.nsw.gov.au; 3 Department of Surgery, St George Hospital, Sydney, NSW 2217, Australia; 4 St George and Sutherland Clinical School of Medicine, University of New South Wales, Sydney, NSW 2052, Australia; 5 Intensive Care Unit, St George Hospital, Sydney, NSW 2217, Australia

**Keywords:** mucus plugs, Bromelain, Acetylcysteine, ventilatory resistance, mucus dissolution, muco-obstructive disease, ovine lung, viscosity

## Abstract

Mucus plugging of the respiratory tract occurs in airway diseases, including asthma, chronic obstructive pulmonary disease, and cystic fibrosis. It can cause blockage of the airways, leading to breathlessness and lung failure. Here, we used a ventilatory setup to demonstrate the effect of BromAc^®^ in dissolving mucus plugs in a novel ex vivo ovine obstructive lung model. Mucus simulant was filled into the trachea of freshly slaughtered ovine lungs and ventilated via an endotracheal tube (ETT) using Continuous Mandatory Ventilation. Predetermined single or repeated doses of Bromelain, Acetylcysteine (Ac), BromAc^®^, and saline control were administered via an Aerogen^®^ vibrating nebulizer and ventilated for 30 or 60 min. Ventilatory recording of resistance, compliance, and tidal volume was conducted, and rheology pre- and post-treatment were measured. A significant decline in airway resistance (*p* < 0.0001) compared to the saline control was observed when treated with Bromelain, Ac, and BromAc^®^, with the latter showing a stronger mucolytic effect than single agents. The decline in resistance was also effective in shorter time points (*p* < 0.05) at lower doses of the drugs. Changes in compliance, peak pressure, and tidal volume were not observed after administration of the drugs. Rheology measurements revealed that BromAc^®^^TM^ significantly reduced the viscosity of the mucin at the end of 30 min and 60 min time points (*p* < 0.001) compared to the saline control. BromAc^®^ showed complete dissolution of the respiratory mucus simulant and improved ventilatory airflow parameters in the ex vivo ovine model.

## 1. Introduction

Mucus plugging and hypersecretion are now considered crucial in several respiratory pathologies, which have the potential to cause death if untreated [1,2]. Respiratory disease is the third among the top ten diseases worldwide (WHO, 2019), with approximately 7.7 million reported deaths each year [3]. Patients with severe acute and chronic respiratory inflammatory diseases, including acute respiratory distress syndrome (ARDS), asthma, COPD, bronchiectasis, and cystic fibrosis, are known to secrete excessive mucus [4,5]. As a result, many hospitalized patients with these respiratory diseases suffer from severe dyspnea and hypoxemia. On average, about 80% of the patients with inflamed lungs secrete these thick, jelly-like mucilaginous substances. As they accumulate in the lung, they thicken and become highly viscous, severely affecting ciliary movement, thus causing respiratory obstruction and distress to patients [6]. Although these patients are often intubated to manage hypoxia, the blockage of their airways by these thick secretions presents a barrier [7,8] to efficient gas exchange, resulting in respiratory failure, barotrauma, and, for many patients, potential mortality [9,10]. 

In pathological airway conditions, an increase in mucus production occurs primarily due to goblet cell hyperplasia or through an increase in their inherent secretory capacity, usually as a response to harmful molecules/chemicals such as cigarette smoke, air pollution, or through severe infectious conditions such as bronchiectasis and cystic fibrosis [11,12,13]. Over time, the increase in mucus hypersecretion severely obstructs airflow in these patients, speeding up the decline in lung function. In addition, the variable lung inflammatory responses to infections and foreign substances further compromise the ciliary mucociliary clearance efficiency and affect the mucus’s biophysical properties [14]. A reduced mucociliary clearance enhances the susceptibility to chronic airway infections, especially opportunistic airway pathogens such as *Pseudomonas aeruginosa* (*PsA*), *Haemophilus influenzae*, and *Staphylococcus aureus*. Mucus accumulation in the trachea and impaired mucus clearance are also observed in intubated patients, particularly in those who are neurologically compromised. Chest physiotherapy is variably effective, and suctioning is a common practice for removing the accumulated mucus; however, limited by access to the proximal airway, repeat bronchoscopy can be a logistical challenge [15]. 

The composition of sputum in samples from patients with asthma, chronic obstructive pulmonary disease, cystic fibrosis, COVID-19, and other respiratory diseases share similarities, containing double-stranded RNA, mucins (mainly MUC1, MUC5AC, and MUC5B) [16], cell debris, and lipids. Bronchoscopy for mucus plugging is frequently required during mechanical ventilation. Recently, Mitja et al. (2022) [17] reported prominent findings of abundant thick secretion in patients with COVID-19, which was challenging to suction in 91% of patients, and muco-hematic plugs required the use of saline and mucolytic agents in 32% of patients. Furthermore, another study described the abundant presence of mucin, especially MUC5B, in the distal airway regions of patients with COVID-19 [17], beyond the reach of bronchoscopy, making the intervention challenging. Therefore, the targeted administration of a potent mucolytic and anti-inflammatory agent may have comprehensive patient management benefits. 

To this end, the mucolytic agent BromAc^®^ (a combination of selective stem bromelain components and Acetylcysteine) is an effective mucin-digesting enzyme and solubilizer for thick mucus. We previously demonstrated the effect of BromAc^®^ in mucinous tumors [18], oncology [19], COVID-19 [20], and cystic fibrosis sputum [21]. Here, we investigated the efficacy of BromAc^®^ using sputum-plugged ovine lungs as a model of mucus-obstructed airways. We simulated an obstructive lung model using artificial mucin. We primarily examined the mucolytic effect of BromAc^®^ in a ventilated ex vivo ovine lung. This study also compared the efficacy of treatments of BromAc^®^ versus Bromelain or Acetylcysteine alone at several time points and concentrations. Furthermore, data on resistance, compliance, peak pressure, tidal volume, and viscosity were collected. 

## 2. Materials and Methods

### 2.1. Drugs

Bromelain (PPP-20-811) was manufactured by Mucpharm Pty Ltd (Sydney, New South Wales, Australia). The 200 mg/mL Acetylcysteine (AC) Lot#90410 was clinical-grade and purchased from Link Pharma (New South Wales, Australia). All other reagents used were purchased from Sigma Aldrich, Sydney, Australia.

### 2.2. Preparation of Drugs (Bromelain, AC, and BromAc^®^)

Separate doses of BromAc^®^, containing 250 µg/mL of Bromelain with 20 mg/mL Acetylcysteine (BromAc^®^ High) and 125 μg/mL Bromelain with 10 mg/mL and 20 mg/mL Acetylcysteine (BromAc^®^ Low1 and 2), were prepared in 0.9% sodium chloride (saline). Bromelain (125 and 250 µg/mL) was also prepared in 0.9% saline, adjusted to pH 7.0, whilst 10 and 20 mg/mL (1.0 and 2.0%) Acetylcysteine were prepared by 20% dilution of the clinical solution (Link Pharma) with 0.9% saline. The drugs were prepared freshly, and their pH adjusted to 7.0. The drugs were stored at 4 °C and equilibrated to ambient room temperature (23 °C) just before use. 

### 2.3. Preparation of Mucus Simulant

Soft mucin extracted from Pseudomyxoma peritonei (PMP) during surgical tumor resection was used to prepare the simulant. It primarily contains mucin, MUC1 and MUC5AC, cellular debris, double-stranded RNA (dsRNA) from degrading cells, lipids, and other cellular materials [19]. The soft mucin was mixed with phosphate-buffered saline (PBS) to obtain a consistency of sticky, sputum-like purulent as in patients with cystic fibrosis [22]. For the experiments, we obtained large quantities of PMP patient mucin (about 2 kg) from patients undergoing cytoreductive surgery. The extracted mucin was aliquoted and stored at −80 °C in 250 mL yellow-capped specimen jars until it was used in the experiment. To maintain the consistency of the sample, all further experiments were conducted using the same thawed aliquots. Briefly, to 250 mL of PMP mucin, approximately 50 mL of PBS was added, homogenized rapidly and continuously several times using a 25 mL serological pipette, and then vortexed until an even flow rate was obtained. The fluid’s viscosity was determined using a rotational digital viscometer (Drawell NDJ-55), and approximately 20,000 millipascal-second (mPa·s)as observed for all experiments [23]. 

### 2.4. Ventilator Settings and Measurements

A Drager Evita XL ventilator was used in the circuit with a size 9.0 endotracheal tube, Aerogen ProX mesh nebulizer, and Fisher & Paykel 850 humidifier connected to medical oxygen and air. Sheep lungs, via the trachea, were connected to the end of the endotracheal tube. The lungs were placed in a water bath at 37 °C. The ventilator was set to continuous mechanical ventilation (CMV) with the following parameters: tidal volume (VT)—0.400 L; inspiratory time—2.0; frequency (f)—9.0 bpm; Pmax—35 cmH2O; positive-end expiratory pressure (PEEP)—0 cmH20. The humidifier Fisher & Paykel 950 (Auckland, New Zealand) was set to Invasive mode at a temperature of 37 °C.

### 2.5. Mucus Simulant Administration and Nebulization of Drugs in Ovine Lung

**Figure 1:**
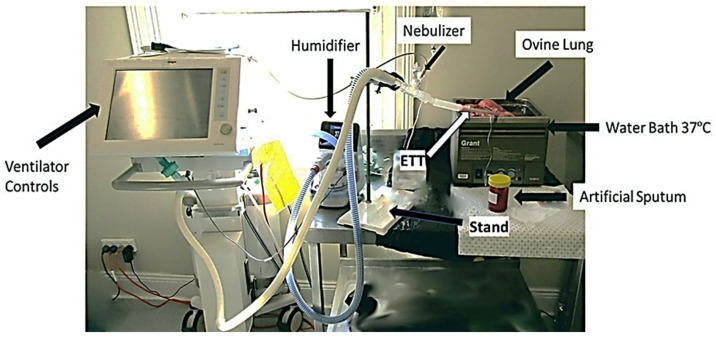
Outlines the equipment and experiment setup used to treat the plugged mucin in the endotracheal tube connected to the ovine lungs. The mucin was inserted into the endotracheal tube (ETT), and the drug was administered through the nebulizer chamber.

Western Sydney Meat Works kindly donated a freshly slaughtered ovine cardiorespiratory system, including the trachea, lungs, and heart. It was stored at 4 °C for immediate use, i.e., within 24–48 h. After a stable ventilatory period, 10 mL of mucus simulant was administered through the endotracheal tube and into the trachea to simulate mucus obstruction (Figure 1). After 1 min of ventilation, nebulization of 5 or 10 mL of saline (control), Bromelain, Ac, or BromAc^®^ commenced.

Ventilator recordings for resistance, compliance, peak pressure, and tidal volume were recorded, controlling for peak pressure and tidal volume. Two experiments were conducted: one for 30 min with a single nebulization of the drugs at the start (0 time point), with ventilator recordings conducted at intervals of 0, 5, 10, 15, and 30 min, and the second experiment for a longer duration of 60 min with recordings obtained at intervals of 0, 15, 30, 45, and 60 min. For the latter experiment, nebulization was performed twice: once at the 0th and the other at the 30th minute. Drugs were administered at concentrations mentioned in the Section 2.2. Three independent studies (new lung) for each experiment were performed. After administration, the mucus from the ovine lung was collected into a 50 mL jar, and viscosity was estimated using a rotational viscometer.

### 2.6. Viscosity Measurements

The viscosity of pretreated (control) and treated mucus was measured using a digital rotational viscometer (Drawell NDJ-5S). All viscosity measurements were carried out at an ambient room temperature (~25 °C), using rotor L3 and L4 spindles at a shear rate between 6 and 60 rpm based on the fluid viscosity at the start and end of the experiment, i.e., at the 0 and 30 or 60 min time points after treatment with 0.9% saline (control), Bromelain, Ac, and BromAc^®^, following the standard measurement protocols described in the instrument’s manual.

### 2.7. Statistical Analysis

All analyses are represented as mean and 95% CI. A two-way ANOVA was performed to analyze the variance across the ventilator parameters, and the comparison between the saline control and drug treatment groups was carried out using Dunnett’s multiple comparison test. The analysis was performed using GraphPad Prism V9.4.1. A *p*-value < 0.05 was considered significant.

## 3. Results

### 3.1. Effect of Bromelain, Ac, and BromAc on Ventilatory Resistance

**Figure 2:**
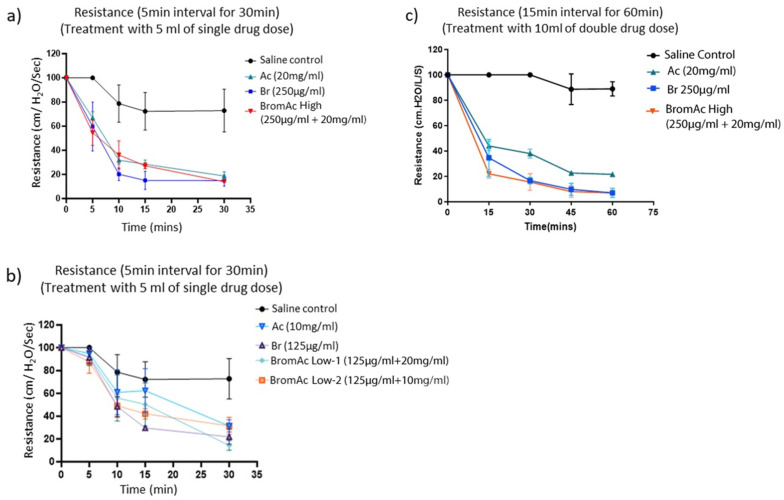

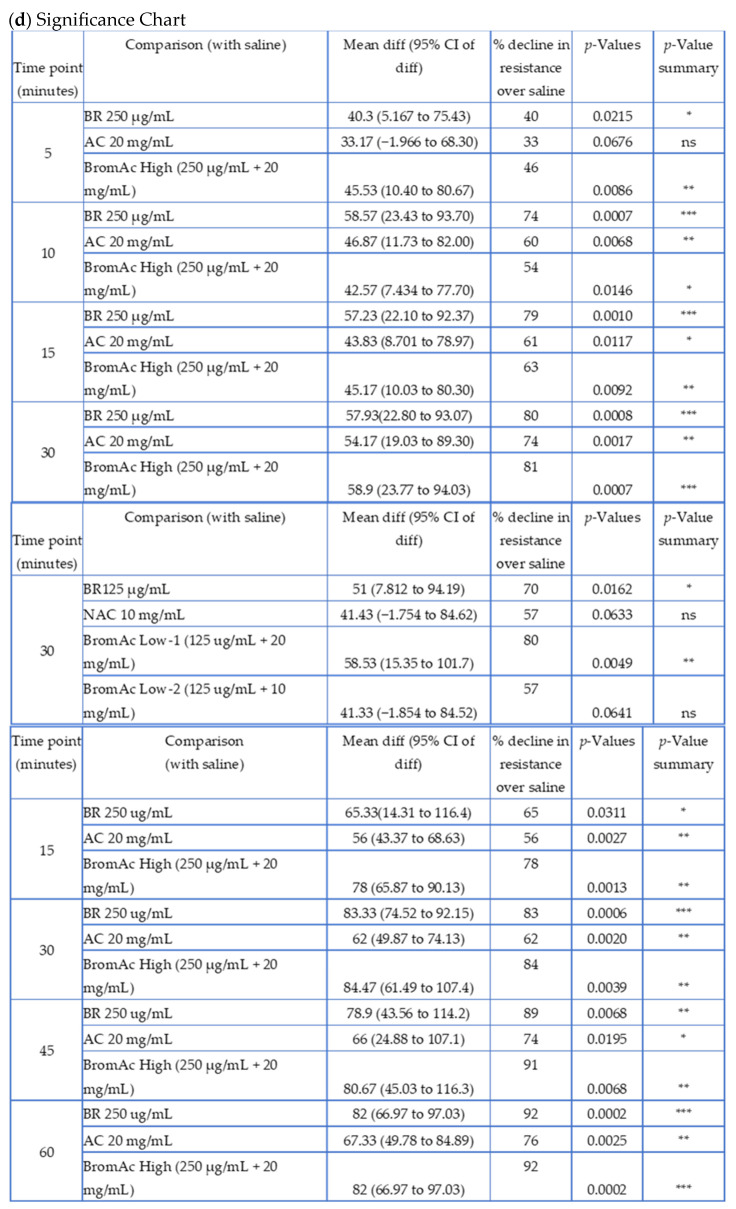
Representative graphs illustrate the decline in airway resistance over time when treated with BromAc, Bromelain, and Ac at (**a**) higher and (**b**) lower doses for 30 treatments (5 min intervals) and (**c**) higher doses for sixty minutes (15 min time intervals). Mean differences, per cent decline in resistance, and *p*-values between the treatment groups and saline control are presented in the (**d**) significance chart. Also, significant changes were only observed at the lower drug concentrations (**b**) at the 30 min time point. ns: non-significant, * *p* < 0.05, ** *p* < 0.01, *** *p* < 0.001.

All drug treatments, independently or in combination, showed a decline in ventilatory resistance compared to the saline control at the thirty- and sixty-minute time points (Figure 2a–c). However, a significantly quicker decline in ventilatory resistance with BromAc^®^ High five minutes after nebulization (*p* < 0.001) was observed. BromAc^®^ also showed a higher percent reduction in resistance than single-agent Bromelain and Acetylcysteine at the five-minute time point. Thirty minutes after administration, Bromelain 250 µg/mL and BromAc^®^ High showed a similarly significant percent (up to 80%) decline in resistance compared to saline, although Ac was lower in percent inhibition than Bromelain alone and BromAc (Figure 2a).

A significant change with lower concentrations of BromAc was noticed only at the 30 min time point after nebulization. BromAc^®^ Low-1 showed the most significant reduction in ventilatory resistance compared to Bromelain, Ac alone, and BromAc^®^ Low-2 (Figure 2b).

Over the 60 min experiment (Figure 2c), airway resistance recovered to 92% with BromAc^®^ High compared to the control, with a similar reading from the high-dose Bromelain alone treatment at 60 min (Figure 2c). More clinically relevant, by the 15 min mark, BromAc^®^ High had decreased resistance compared to all agents alone, indicating it was the most effective in this experiment.

### 3.2. Effect of Bromelain, Ac, and BromAc on Ventilatory Compliance, Tidal Volume, and Peak Pressure

**Figure 3:**
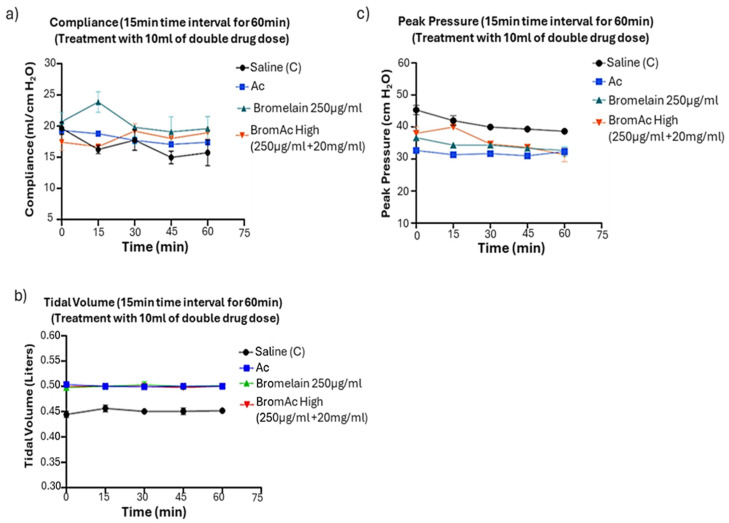
Representative graphs demonstrating the ventilation parameters. No significant changes in (**a**) compliance, (**b**) tidal volume, and (**c**) peak pressure were observed over time when treated with higher doses of BromAc^®^ High, Bromelain, and Ac for sixty minutes (15 min intervals).

Limited by the model used in this experiment, we did not observe a significant change in compliance values at lower and higher drug concentrations for the 60 min time-point experiments (Figure 3a).

No tidal volume or peak pressure change was observed between the 60 min time point and zero across treatment groups compared to the saline control (Figure 3b,c).

### 3.3. Effect of Bromelain, Acetylcysteine, and BromAc^®^™ on Mucus Viscosity Using Rheology

**Figure 4:**
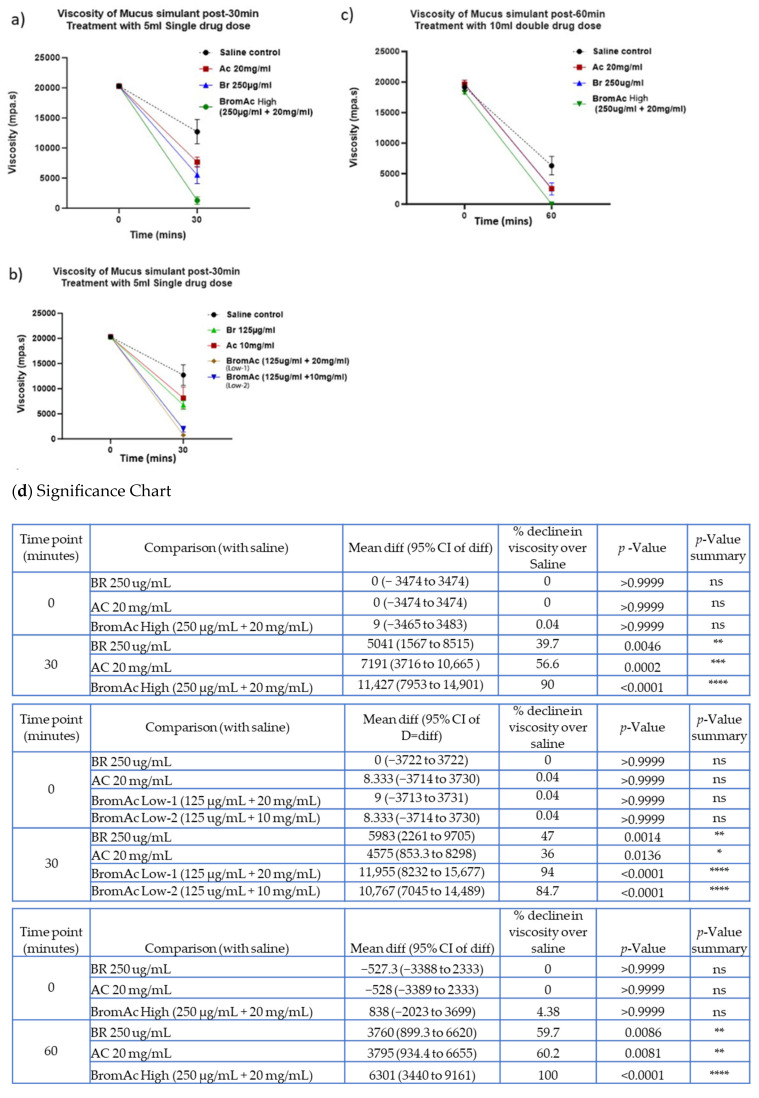
Representative graphs show a decline in viscosity when treated with BromAc, Bromelain, and Ac at (**a**) a higher and (**b**) lower dose for 30 min and (**c**) a higher dose for sixty minutes. Mean differences, percent change, and *p*-values between the treatment groups and saline control are presented in the (**d**) significance chart. ns: non-significant, * *p* < 0.05, ** *p* < 0.01, *** *p* < 0.001 and **** *p* < 0.0001.

We observed a significant decline in the viscosity of mucus irrespective of concentration or time point. The greatest potency was observed with BromAc^®^ High, which significantly reduced the viscosity measurement (1281.4 m.pas) compared to the saline control (12,708.5 mpa.s) (*p* < 0.0001) by the end of 30 min (Figure 4a). BromAc^®^ High also showed a greater viscosity reduction compared to Bromelain and Ac alone treatments relative to the saline control (Figure 4a,d). The BromAc^®^ Low-1 treatment also showed a significant decline in viscosity by 30 min, higher than that of BromAc^®^ Low-2 and Bromelain and Ac alone treatment (Figure 4b).

Similarly, at the 60 min time point, high-dose BromAc^®^ dissolved the mucin entirely, with the viscosity reduced to 0% (*p* < 0.0001) vs. the saline control (Figure 4c) and was also observed to be better than the Bromelain and Ac alone treatment.

### 3.4. Viscosity and Ventilatory Parameters and Relationship in the Treatment Group and Control

**Figure 5:**
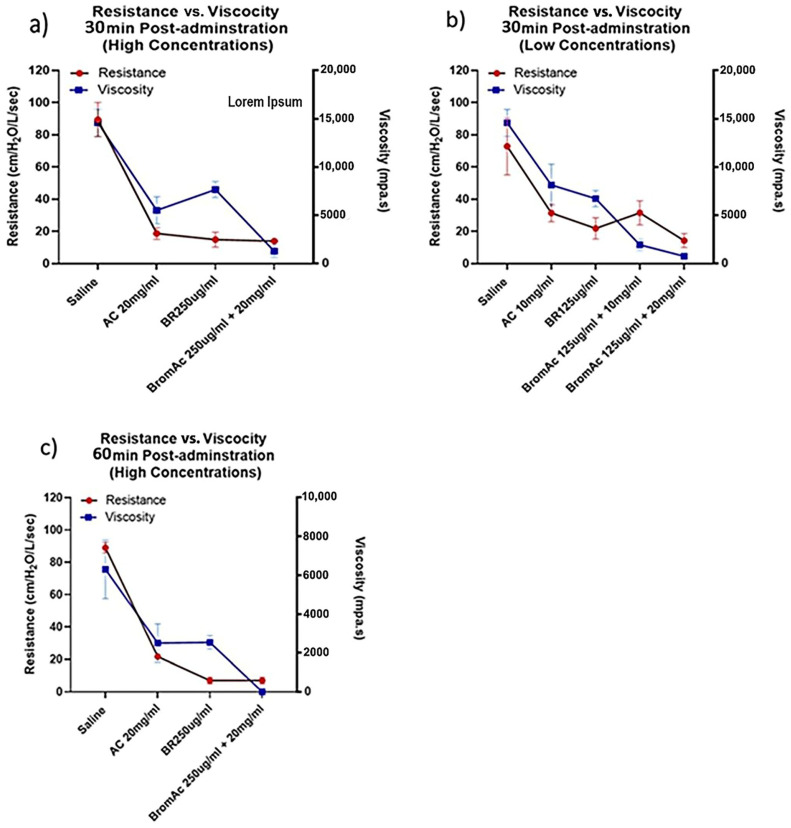
Graphical analysis showing the association between viscosity and resistance post-treatment with Bromelain, Ac, BromAc and saline control at higher doses after (**a**) 30 and (**c**) 60 min and (**b**) at a lower dose for 30 min.

We observed a direct relationship between the decline in resistance and viscosity, with the best outcomes seen in BromAc^®^ compared to single agents or the control (Figure 5a–c).

## 4. Discussion

In the present study, we simulated a respiratory distress model by plugging the ovine lung’s airway with artificial mucus material (simulant) via an endotracheal tube connected to sheep lungs. We delivered saline, Bromelain, Acetylcysteine, and BromAc^®^ using a standard mechanical ventilatory circuit.

Bromelain, an extract from the stem or fruit of the pineapple plant (*Ananas comosus*), consists of many enzymes such as cysteine proteases, peroxidases, phosphatases, and cellulases [24] and has an array of therapeutic properties, including the ability to solubilize mucinous materials [25,26]. Its mucolytic property has been ascribed to the hydrolysis of peptide and glycosidic linkages in polymeric mucin [27]. Acetylcysteine is a sulfhydryl group donor, potentiating the selected enzymes in Bromelain, a reducing agent (antioxidant), and is used as a mucolytic in respiratory diseases since it disintegrates the disulfide bonds interlinking the mucin polymer [28]. BromAc^®^ selectively combines these two agents and has shown great success as a mucolytic for treating the rare tumor of the appendix, pseudomyxoma peritonei (PMP), in a phase 2 evaluation [18]. A novel synergistic finding was the combination of cysteine–protease, which breaks peptide bonds, prolines, and disulfide bond breakers.

In our 30 min and 60 min time-point studies using BromAc^®^, we identified a significant decline in resistance. Interestingly, the effect was seen within the first five minutes after nebulization, with resistance declining by 50%. By 60 min, the resistance was close to a clear airway. Furthermore, the reduction in resistance was directly associated with the reduced viscosity of the mucus, which was seen to be the best in BromAc^®^™ compared to single agents. In muco-obstructive lung diseases such as asthma, COPD, and cystic fibrosis, or in patients under prolonged ventilation, increased accumulation of mucus in the airway is problematic [29]. Mucus in these patients is thicker, tenacious, and not quickly cleared by the normal ciliary and lung. It also harbors opportunistic pathogens, leading to chronic lifetime inflammation, biofilms, and frequent exacerbations. Thick mucus causes non-contractile airway narrowing. All these phenomena contribute to reduced lung airflow [30]. Removing these compact mucus plugs is critical to improving the overall condition of these patients. In addition, a clinical intervention that targets biofilms and reduces inflammation is required.

With rheology, there was a significant decline in the viscosity of BromAc^®^ mucin, which was indicated by a sharp drop even at the shorter time points; however, BromAc^®^™, Bromelain, and Acetylcysteine all showed almost similar viscometry readings at the 60 min endpoints. Prolonged exposure to the agents at 60 min may have contributed to the similar effect seen among all agents. In the clinical setting, absorption and clearance of the drug are expected to be rapid, making the shorter time points more clinically significant. The drop in viscosity may correlate directly with reduced airway resistance and tidal volume [31]. Saline also showed a drop in viscosity compared to baseline, which could suggest the possible action of monovalent sodium ions [28] or hydration [29] in reducing mucin viscosity.

In the current respiratory model of obstructed or restricted airways by mucinous plugs, the sheep lung model does not fully represent human lungs because of the absence of the chest wall, diaphragm, and perfusion. We used post mortem ovine lungs that could have undergone morphological changes, affecting their elastic properties. All ventilator parameters, particularly compliance, were therefore impacted. We attempted to control this by placing the lungs in a water bath. There was a clear difference in the modulation of some parameters, such as airway resistance and viscosity, indicating the clinically feasible solubilization capability of BromAc^®^™. Additional research into the effect of therapeutic solutions at shorter intervals after administration may provide clinically significant outcomes for obstructed airways.

The passage of air and oxygenation is hampered by thick mucinous secretions in response to airway disease and inflammatory response, providing a highly effective barrier and environment for pathogens, which may contribute to alveoli collapse [7,8]. Patients on mechanical ventilation may suffer from lung damage due to the higher pressures required to keep the alveoli open and maintain oxygenation [32,33]. Clearance of a mucinous barrier may provide more efficient gas exchange, limiting pressure [34] and nosocomial infection. Furthermore, multiorgan failure following respiratory failure is a known complication in patients with ARDS and COVID-19 [5,35]. Many patients with asthma have mucinous casts blocking the airway in addition to constriction. Mucus plugs have a long-term effect on airflow in ambulant patients, associated with a decline in lung function.

A clinical phase 1 trial has been completed with nebulized BromAc^®^ at both concentrations utilized in this ex vivo study, with results supporting expansion to clinical evaluation in patients with muco-obstructive respiratory diseases. We are currently conducting preclinical safety testing in animal models, wherein various doses of nebulized BromAc are being tested. Also, BromAc efficacy in an ovalbumin-induced asthma model is under evaluation.

## 5. Conclusions

We demonstrated ex vivo that BromAc^®^ is a highly effective agent with the potential for dissolving the mucus plugging associated with chronic and acute airway diseases. BromAc^®^™, possibly through its mucolytic properties, also showed improved ventilatory parameters. The clinical evaluation of BromAc^®^ aims to establish its utility in managing muco-obstructive lung diseases in a hospital setting.

## Data Availability

The original contributions presented in the study are included in the article, further inquiries can be directed to the corresponding author.
